# Intestinal helminth infections among inmates in Bedele prison with emphasis on soil-transmitted helminths

**DOI:** 10.1186/s13104-015-1775-7

**Published:** 2015-12-14

**Authors:** Bahiru Terefe, Endalew Zemene, Abdurehman E. Mohammed

**Affiliations:** Jimma University Specialized Hospital Laboratory, Jimma University, Jimma, Ethiopia; Department of Medical Laboratory Sciences and Pathology, College of Health Sciences, Jimma University, Jimma, Ethiopia

**Keywords:** Intestinal helminths, Inmates, Ethiopia

## Abstract

**Background:**

Intestinal helminths infect more than two billion people worldwide. They are common in developing countries where sanitary facilities are inadequate. There is scarcity of documented data on the magnitude of intestinal helminths among inmates in Ethiopia. The aim of this study was to determine prevalence of intestinal helminth infections among inmates in Bedele prison, south-western Ethiopia.

**Methods:**

A cross-sectional study involving 234 inmates in Bedele prison was conducted in April 2012. Socio-demographic data was collected from each study participant using semi-structured questionnaire. Fresh stool specimens were collected and processed using modified McMaster technique.

**Results:**

At least one species of intestinal helminth was identified in 111 (47.4 %) of the inmates. *Ascaris lumbricoides* was the most predominant parasite isolated, followed by the hookworms. Most of the cases of soil-transmitted helminths (STHs) were light infections. Untrimmed hand fingernails was significantly associated with *A. lumbricoides* infection (AOR 0.383, 95 % CI 0.200–0.731).

**Conclusion:**

Intestinal helminths are common among the inmates in Bedele prison. Health information should be given to the inmates on proper personal hygiene practices with emphasis on trimming of hand fingernails. Monitoring helminth infections in the inmate population is required.

## Background

Intestinal helminth infections are among the most common infections in developing countries. The soil-transmitted helminths (STHs) are wide spread in areas where access to sanitary facilities is limited. Globally, an estimated 4.5 billion individuals are at risk of STH infections. The STHs *Ascaris lumbricoides, Trichuris trichiura* and the hookworms (*Ancylostoma duodenale* and *Necator americanus*) infect an estimated 819.0 million, 464.6 million and 438.9 million people worldwide, respectively. The vast majority of the STH infections occur in Asia, sub-Saharan Africa and the Americas [1, 2].

Soil-transmitted helminths impair health of the infected individuals in several ways. These include impact on digestion and absorption of foods [3], physical fitness and loss of appetite [4], cognitive function [5], school performance and attendance [6, 7]. In adults, they are associated with reduced productivity and work capacity [6]. The STHs pose huge disease burden, majority of the disease burden being due to the hookworms [1].

Determining local magnitude of STHs and assessment of associated risk factors helps targeted intervention activities to be carried out. In Ethiopia, several epidemiological studies have been conducted on STHs, often focusing on preschool and school children [8–11]. Most of these studies reported high prevalence of STHs. Studies on magnitude of STHs in inmates in Ethiopia are scarce; while various prevalence rates were reported in some developing countries [12–14]. Inmates in developing countries are likely vulnerable to infectious diseases as a result of overcrowding and inadequate health care services. Although helminth infections are very common in Ethiopia, the magnitude of infection is not well explored in the inmate population. Scarcity of published reports on magnitude of STH infections in inmates in Ethiopia initiated us to carry out this study.

## Methods

### Study setting

A cross sectional study was conducted to determine prevalence and associated risk factors of STHs in inmates in Bedele prison in April 2012. The town is located 483 kms south-west of the capital Addis Ababa. Administratively, the town is divided into four *kebeles* (smallest administrative units in Ethiopia). The district is predominately rural and the livelihood of the inhabitants mainly depends on subsistent farming. According to the 2007 Central Statistical Agency census report, the projected total population of the district is 139,425 [15]. During the study period, the prison houses a total of 600 inmates. In the town, there was one district hospital recently established, one health centre and several private clinics. Located at average altitude of 2000 m above sea level, Bedele town has temperature ranging from 20 to 28 °C.

### Sample size and sampling technique

Sample size for the study was determined using the general formula for single population proportion, assuming: expected prevalence (p) of 50 % at 95 % confidence level. Since the source population were a total of 600 inmates during the study period, finite population correction formula was utilized. Finally, the sample size calculated was a total of 234 individuals. Study participants were selected by systematic sampling from list of the inmates.

### Data collection

Data on socio-demography and predisposing factors were collected using semi-structured questionnaire. The questionnaire was first prepared in English and translated to the local language (*Afan Oromo*). The questionnaire addressed socio-demographic information and personal hygienic practices including: hand fingernail trimming status and hand washing practice before meal among others.

Moreover, single, fresh stool specimen was collected from each study participant and processed by modified McMaster technique for faecal egg count as described earlier [16]. Briefly, 2 g fresh stool samples were homogenized in 30 ml of floatation solution (saturated sodium chloride solution) and poured through mesh three times. The faecal debris was squeezed after the samples were sieved for the third time. The two chambers of McMaster slide were filled with aliquot of the sample (2 × 0.15 ml), and eggs of each STH encountered were counted using 100× magnification of the microscope. Finally, the egg per gram (epg) of stool of each STH was obtained by multiplying the total number of STH eggs counted by 50.

### Data analysis

Infection intensity class of the STHs was determined by faecal egg count threshold set by the World Health Organization [17]. Accordingly, infection of *A. lumbricoides* was considered light, moderate and heavy when the egg count was 1–4999, 5000–49,999 and >49,999 epg, respectively. *T. trichiura* infection was considered light, moderate and heavy when the egg count was 1–999, 1000–9999 and >9999 epg, respectively. The classification for hookworm infection intensity was 1–1999, 2000–3999 and >3999 epg for light, moderate and heavy infections, respectively.

Data were entered, cleaned and analyzed using SPSS version 18.0 statistical software package. Descriptive statistics (frequency, mean and range) were used to summarize the socio-demographic profile of the study participants. Bivariate and multivariate logistic regressions were utilized to assess association of the outcome helminth infection with independent variables. The independent variables included in the model include gender, educational status, residence and occupation before imprisonment, duration of stay in the prison, trimming pattern of hand fingernails and hand washing pattern before meal. p values less than 0.05 were considered statistically significant.

### Ethical clearance

The study was ethically approved from Department of Medical Laboratory Sciences and Pathology, Jimma University. Permission was obtained from the prison administration after explaining purpose of the study and procedures of data collection. Written consent was obtained from each study participant before data collection. Inmates who were positive for intestinal helminth(s) were treated for free according to the national guideline.

## Results

### Description of the study participants

The study included 234 inmates (94.4 % male and 5.6 % female). Age of the study participants ranged from 13 to 80 years (mean age 30.84 years). Most (71.8 %) of the inmates were farmers before imprisonment. Majority (90.6 %) of the inmates responded stay in the prison for less than 2 years. Socio-demographic profile of the study participants is presented in Table 1.

**Table 1:** Distribution of helminth infection with socio-demographic characteristics among inmates in Bedele prison, April 2012

Demographic characteristic	Frequency (%)
Age group in years	
10–19	56 (23.9)
20–29	80 (34.2)
30–39	53 (22.6)
40–49	19 (8.1)
>50	26 (11.1)
Gender	
Male	221 (94.4)
Female	13 (5.6)
Educational status	
Illiterate	88 (37.6)
Literate	146 (62.4)
Residence before imprisonment	
Urban	51 (21.8)
Rural	183 (78.2)
Duration in the prison	
≤2 years	212 (90.6)
>2 years	22 (9.4)
Occupation before imprisonment	
Farmer	168 (71.8)
Merchant	16 (6.8)
Students	32 (13.7)
Others	18 (7.7)

### Prevalence and intensity of helminth infections

The overall prevalence of helminth infection was 47.4 %. Five species of intestinal helminths were identified. The most predominant helminth encountered in this study was *A. lumbricoides,* which accounted for 42.6 % of the helminths identified. Forty-eight (32.4 %) and 32 (21.6 %) of the helminths identified were the hookworms and *T. trichiura,* respectively. *Enterobius vermicularis* and *Hymenolepis nana* were identified in four and one inmate, respectively.

Thirty-three of the helminth infected individuals (29.7 %) had multiple helminth infections. The combination of *A. lumbricoides* and *T. trichiura* dominated the cases of multiple infections, followed by *A. lumbricoides* and the hookworms. Moreover, four individuals were infected with *A. lumbricoides*, *T. trichiura* and the hookworms.

Intensity of STH infections among the inmates is presented in Table 2. Most of the study participants with STH infection in this study had light infections. However, 34.8 % of the study participants infected with *A. lumbricoides* had moderate infections. There was also a case of heavy hookworm infection.

**Table 2:** Intensity of *A. lumbricoides*, *T. trichiura* and hookworm mono-infections among inmates in Bedele prison, April 2012

Intensity of infection	Soil-transmitted helminths		
	*A. lumbricoides* n (%)	*T. trichiura* n (%)	Hookworms n (%)
Light	23 (74.2)	12 (100)	29 (96.7)
Moderate	8 (25.8)	0 (0)	0 (0)
Heavy	0 (0)	0 (0)	1 (3.3)
Geometric mean (EPG)	849	220	372
Range	100–24,500	50–650	50–4150
Total	31	12	30

### Factors associated with helminth infections

Most of the study participants (78.2 %) responded residence in rural area before imprisonment and the remaining 21.8 % responded urban residence, with intestinal helminth prevalence rate of 47 and 49 %, respectively. The difference in intestinal helminth prevalence among inmates from rural and urban areas was not significant. Prevalence of intestinal helminths among illiterate and literate inmates was 50 and 45.9 %, respectively. Similarly, the difference in prevalence of intestinal helminths among illiterate and literate inmates was not significant. Moreover, no significant difference in intestinal helminth prevalence was observed between male and female study participants (Table 3).

**Table 3:** Univariate analyses of factors associated with intestinal helminth infection among inmates in Bedele prison, April 2012

Variables	Intestinal helminth		COR (95 % CI)
	Positive n (%)	Negative n (%)	
Gender			
Male	102 (46.2)	119 (53.8)	1
Female	9 (69.2)	4 (30.8)	0.381 (0.114–1.274)
Educational status			
Illiterate	44 (50.0)	44 (50.0)	1.179 (0.694–2.003)
Literate	67 (45.9)	79 (54.1)	1
Residence before imprisonment			
Urban	25 (49.0)	26 (51.0)	0.922 (0.496–1.716)
Rural	86 (47.0)	97 (53.0)	1
Occupation before imprisonment			
Farmer	81 (48.2)	87 (51.8)	0.752 (0.356–1.587)
Student	16 (50.0)	16 (50.0)	0.700 (0.265–1.852)
Others	14 (41.2)	20 (58.8)	1
Duration of stay in the prison			
≤2 years	106 (50.0)	106 (50.0)	0.294 (0.105–0.826)*
>2 years	5 (22.7)	17 (77.3)	1
Fingernail status			
Trimmed	36 (35.0)	67 (65.0)	1
Untrimmed	75 (57.3)	56 (42.7)	0.401 (0.235–0.684)*
Hand washing before meal			
Always	101 (46.3)	117 (53.7)	1
Sometimes	10 (62.5)	6 (37.5)	0.518 (0.182–1.475)

* Significant at p < 0.05

On the other hand, more than half (56.0 %) of the study participants had untrimmed hand fingernails at the point of data collection. Of these, 75 (57.3 %) were positive for intestinal helminths. There was significantly higher prevalence of intestinal helminths among inmates with untrimmed hand fingernails (COR 0.401, 95 % CI 0.235–0.684) compared to inmates with trimmed fingernails. Moreover, prevalence of intestinal helminths among inmates within the first two years of prison was significantly higher than the prevalence among inmates who served more than two years (COR 0.294, 95 % CI 0.105–0.826) (Table 3).

Thirty-seven (15.8 %) of the study participants responded that they do not wear shoes at all. Of these, 21.6 % were positive for the hookworms. In those who responded to wear shoes (84.2 %), prevalence of hookworm was (11.2 %). Prevalence of hookworm infection did not show significant difference between inmates who do not wear shoes and those who wear shoes.

After adjusting for other variables, no single predictor of intestinal helminth infection was found in this study. However, untrimmed hand fingernails was significantly associated with *A. lumbricoides* infection (AOR 0.383, 95 % CI 0.200–0.731) (Table 4).

**Table 4:** Factors associated with *A. lumbricoides* infection among inmate in Bedele prison, April 2012

Characteristics	*A. lumbricoides* ^a^		COR (95 % CI)	AOR (95 % CI)
	Positive n (%)	Negative n (%)		
Gender				
Male	57 (25.8)	164 (74.2)	1	1
Female	6 (46.2)	7 (53.8)	0.405 (0.131–1.257)	0.355 (0.107–1.179)
Educational status				
Illiterate	41 (28.1)	133 (71.9)	1.171 (0.641–2.140)	0.994 (0.498–1.986)
Literate	22 (25)	66 (75)	1	1
Residence before imprisonment				
Urban	15 (29.4)	36 (70.6)	1.172 (0.590–2.328)	0.896 (0.345–2.328)
Rural	48 (26.2)	135 (73.8)	1	1
Occupation before imprisonment				
Farmers	42 (25)	126 (75)	1.250 (0.553–2.827)	1.117 (0.355–3.511)
Students	11 (24.4)	21 (65.6)	0.795 (0.282–2.245)	0.620 (0.191–2.010)
Others	10 (29.4)	24 (70.6)	1	1
Duration of stay in the prison				
≤2 years	58 (27.4)	154 (72.6)	0.781 (0.276–2.214)	1.091 (0.364–3.267)
>2 years	5 (22.7)	17 (77.3)	1	1
Finger nail status				
Trimmed	18 (17.5)	85 (82.5)	1	1
Untrimmed	45 (34.4)	86 (65.6)	0.405 (0.217–0.755)**	0.383 (0.200–0.731)**
Hand washing before meal				
Always	58 (26.6)	160 (73.4)	1	1
Sometimes	5 (23.5)	11 (76.5)	0.798 (0.266–2.393)	0.843 (0.268–2.649)

** Significant at p < 0.05

^a^Includes both *A. lumbricoides* mono-infection and multiple infection with other intestinal helminths

## Discussion

The major aim of this study was to determine the prevalence of intestinal helminths among inmates in Bedele prison. Accordingly, 47.4 % of the inmates were found to be infected with at least one species of intestinal helminth. The prevalence of intestinal helminths obtained in this study is slightly lower than the prevalence recently reported from Shewa Robit [18]. At least one species of intestinal helminth was reported in 58.7 % of the inmates in Showa Robit prison in Ethiopia. Compared to a study done on the general population in south-west Ethiopia [19], in which more than half of the studied population had been found to harbour intestinal helminths, the prevalence in this study is lower. Contrary to the prevalence of intestinal helminths reported in a study done among mothers in Butajira in Ethiopia [20] and inmates in Owerri prison [14], the prevalence obtained in this study is slightly higher.

Despite the lower prevalence of intestinal helminthic infections compared to most of the studies, nearly half the inmate population harboured at least one species of the parasite. This calls for targeted interventions to be carried out to prevent infection of the inmates with the intestinal nematodes. Although the impact of STHs is remarkably higher in children [21], they may also affect productivity of the inmates [22]. *Ascarislumbricoides* is the most common STH in Ethiopia. In this study it was detected in 26.9 % of the inmates. This magnitude is lower than an earlier report of 40.9 % prevalence among urban dwellers in Jimma [19], but higher than the study done in Butajira (8.8 %) [20]. The prevalence of hookworm infection in this study (20.5 %) is comparable with the one reported in the previous study in Jimma town (17.5 %). The STHs and other neglected tropical diseases (NTDs) are common in Ethiopia. It is estimated that Ethiopia is among the top three countries to have the highest number of NTD cases [23].

To identify factors associated with STH infections, bivariate and multivariable analyses were carried out. Bivariate analysis revealed that untrimmed hand fingernails and duration of imprisonment of less than 2 years were significantly associated with intestinal helminth infections. Transmission of intestinal parasites in general is effected by direct or indirect contamination with human faeces. Untrimmed fingernails are difficult to clean and may harbour parasites stages in the dirt [24]. Positive association of untrimmed fingernails with intestinal helminth infection was reported elsewhere [25]. However, in this study, no single variable was identified as predictor of intestinal helminth infection among the inmates. It has to be considered as a limitation that the risk factors of intestinal helminth infections assessed in this study are not exhaustive, and further study should consider all the possible risk factors of STH infection.

Prevalence of hookworm among inmates who responded wearing shoes and those who responded not wearing shoes was similar. Contrary to this, a previous study done in south-west Ethiopia reported a significant protective role of wearing shoes to hookworm infection [26]. The types of shoes the inmates wear, which was not explored in this study, may account for the difference.

Majority (65.8 %) of the study participants were within their first year of prison term. It had been observed that prevalence of STHs was higher among inmates who were in their first 2 years in the prison compared to those who served for more than 2 years. The difference was significant by the bivariate analysis. It appears that most of the inmates could be infected before imprisonment. Our study is limited by lack of comparative group. Hence, it is not possible to conclude whether the inmates were at a significantly higher risk of intestinal helminth infection compared to the general population.

Majority of the cases of the STHs in this study were light and moderate infections, which is in agreement with most other studies [27, 28]. Soil-transmitted helminth infections tend to be aggregated, with smaller proportion of individuals harbouring high infection intensity.

## Conclusion

Nearly half of the inmates in Bedele prison were infected with at least one species of intestinal helminth. *Ascaris lumbricoides* was the most common STH detected among the inmates, and it was positively associated with untrimmed fingernails. Creating awareness on the modes of transmission of intestinal helminths to the inmate population is recommended. Moreover, regular parasitological examination of stool samples of inmates in Bedele prison is required.
